# Microsatellite Instability-High and High Tumor Mutation Burden Frequencies in Urologic Malignancies Off-Label for Immune Checkpoint Inhibitors in Japan: A Retrospective Single-Institutional Cohort

**DOI:** 10.7759/cureus.69366

**Published:** 2024-09-13

**Authors:** Hiroshi Yaegashi, Kouji Izumi, Tomoyuki Makino, Renato Naito, Hiroaki Iwamoto, Shohei Kawaguchi, Kazuyoshi Shigehara, Takahiro Nohara, Atsushi Mizokami

**Affiliations:** 1 Department of Integrative Cancer Therapy and Urology, Kanazawa University Graduate School of Medical Science, Kanazawa, JPN

**Keywords:** high tumor mutation burden, immune checkpoint inhibitor, microsatellite instability-high, pembrolizumab, urologic malignancies

## Abstract

Objectives

Microsatellite instability-high (MSI-H) and high tumor mutation burden (TMB-high) frequencies were investigated to determine the efficacy and adverse events of pembrolizumab in patients with urologic malignancies (or their equivalents) for which immune checkpoint inhibitors (ICIs) are not covered by Japanese insurance.

Methods

Between February 2019 and April 2024, patients with urologic malignancies (or their equivalents) treated in our department for whom ICIs were not approved by Japanese insurance were screened with an MSI companion diagnostic kit or comprehensive genomic profiling (CGP). The efficacy of pembrolizumab therapy, presence of adverse events, and outcomes were evaluated retrospectively in patients with MSI-H or TMB-high.

Results

In total, 44 patients were tested, and the median age at testing was 70 years. Castration-resistant prostate cancer (CRPC) was the most common (n = 31). Overall, 49 tests were performed, including 22 MSI companion diagnostic kits and 27 CGP tests. Of the 49 tests, 1 detected MSI-H, 2 detected TMB-high, and 1 detected simultaneous MSI-H/TMB-high, with detection rates of 4.1% and 11.1% for MSI-H and TMB-high, respectively. A patient with MSI-H CRPC and neuroendocrine differentiation achieved a complete response and a prolonged duration of response to pembrolizumab without adverse events. The duration of response to pembrolizumab was shorter in a patient with TMB-high, and a CRPC patient with simultaneously detected MSI-H/TMB-high had to discontinue pembrolizumab early due to immune-related adverse events.

Conclusions

Despite the potential benefit of pembrolizumab, MSI-H or TMB-high was less frequently detected in urologic malignancies for which ICIs are not covered by Japanese insurance.

## Introduction

In recent years, in addition to the previously used cytotoxic agents and molecularly targeted therapies, immune checkpoint inhibitors (ICIs) have been added to the treatment repertoire for various cancers, and ICIs are also used as standard treatments in international guidelines. ICIs exert antitumor effects by exposing cancer cells to attack by the immune system. Cancers with accumulated genetic mutations are considered highly immunogenic because of the high expression of abnormal proteins known as neoantigens [1].

ICI therapy is more likely to be successful in patients with mismatch repair gene deficiency (dMMR). In an international phase 2 trial, pembrolizumab was administered to a total of 41 patients divided into 3 groups: dMMR colorectal cancer, other cancers with dMMR, and mismatch repair gene (MMR)-normal colorectal cancer, and showed that pembrolizumab was effective in dMMR cancer regardless of the primary organ [2]. Furthermore, in patients with unresectable or metastatic solid tumors who have a history of treatment, a tumor mutation burden-high (TMB-high) of ≥10 mutations per megabase (mut/Mb) was significantly associated with a clinically objective response rate to pembrolizumab [3]. Based on the results of these studies, after U.S. Food and Drug Administration (FDA) approval, pembrolizumab was approved for use in Japan for microsatellite instability-high (MSI-H) in December 2018 and TMB-high in February 2022.

In sporadic urologic cancers without Lynch syndrome, the frequency of MSI-H is 10%-26% in renal pelvis and ureteral cancer, 3%-28% in bladder cancer, and <10% in localized prostate cancer [4,5]. In addition, the presence or absence of MMR in 12,019 patients with 32 solid tumors was examined, and the results reported that dMMR was present in 24 cancer types, that is, approximately 3% of prostate cancers among urologic cancers and 4% of neuroendocrine tumors among endocrine cancers [6].

Under these circumstances, no studies have presented data on the detection frequency of MSI-H or TMB-high in “urologic malignancies or equivalent tumors for which pembrolizumab is not indicated by Japanese insurance” (other than renal cell carcinoma and urothelial carcinoma) and the therapeutic efficacy and safety of pembrolizumab therapy. The present study is aimed to identify the detection rates of MSI-H and TMB-high in urologic malignancies, evaluate the response to pembrolizumab in these cases, and discuss the implications for clinical practice in Japan.

## Materials and methods

Patients with urologic malignancies (or their equivalents) treated in our department between February 2019 and April 2024, for whom ICI therapy was not approved by Japanese insurance, were screened using MSI companion diagnostics or comprehensive genomic profiling (CGP). The types of urological malignancies for which ICI therapy has been approved by Japanese insurance during the observation period include renal cell carcinoma and urothelial carcinoma. Therefore, patients with a history of these malignancies were excluded. Cases in which MSI was denied by MSI companion diagnostics but CGP was performed were also included in the analysis. The microsatellite stability status was investigated using an approved in vitro diagnostic kit (MSI‐IVD kit; FALCO biosystems, Kyoto, Japan). CGP was either FoundationOne® CDx (F1CDx; Foundation Medicine, Cambridge, MA, USA) or the OncoGuide™ NCC Oncopanel System (NOP; Sysmex Corporation, Hyogo, Japan), whichever was agreed upon by the cancer expert panel, a molecular tumor board composed of multidisciplinary specialists [7]. Our hospital has been approved by the Japanese Ministry of Health, Labour and Welfare as a core hospital for cancer genomic medicine, which allows us to hold our cancer expert panel. Cases that appear to be borderline malignant were also included for consideration based on the consensus of this molecular tumor board.

The MSI-IVD kit is able to detect MSI-H status with DNA isolated only from tumor tissue (without the need for a corresponding blood sample) on the basis of multiplex polymerase chain reaction (PCR) fragment analysis with five mononucleotide repeat markers that were designed by Promega and which have a low susceptibility to genetic polymorphisms [8]. F1CDx is a companion diagnostic test approved by the U.S. FDA to identify patients who are likely to benefit from treatment based on approved therapeutic product inserts for 28 drug therapies and is a next-generation sequencing (NGS)-based CGP technology to examine 324 oncogenes in solid tumors [9]. Meanwhile, NOP is a hybridization capture-based NGS assay designed to examine mutations, amplifications, and homozygous deletions of the entire coding region of 114 genes of clinical or preclinical relevance, along with rearrangements of 12 oncogenes included in the panel [10].

Using a full panel of five markers, tumors showing two or more microsatellite instability markers were classified as MSI-H, tumors showing one instability marker as MSI-L, and tumors showing no instability marker as MSS [8], of which only MSI-H was considered for the present study. For the detection of MSI-H or TMB-high using CGP, the criteria defined in the respective kits were followed [9,10].

If a patient had a positive MSI-IVD kit, MSI-H detection by CGP, or TMB ≥10 mut/Mb, pembrolizumab therapy (200 mg/body, repeated every 3 weeks, or 400 mg/body, repeated every 6 weeks if the disease was deemed stable by the attending physician) was considered. The efficacy, presence of adverse events, and outcome of pembrolizumab therapy were retrospectively evaluated in patients with MSI-H or TMB-high. Pembrolizumab-related adverse events were evaluated using the Common Terminology Criteria for Adverse Events (CTCAE) version 5.0.

If CGP detected a genetic mutation other than MSI-H/TMB-high and a corresponding insurance-approved drug is available, the decision to use pembrolizumab immediately was at the discretion of the attending physician.

Although this study was initially conducted only for MSI testing using only the MSI-IVD kit in Japan, after the expansion of the indication for pembrolizumab in MSI-H and TMB-high detected by CGP, the study had also included MSI-H/TMB-high detected by CGP.

The updated content has been approved by the Kanazawa University Ethical Review Committee (ID 3745-1; Date of approval 21 July 2021).

## Results

A total of 44 cases were evaluated, including duplicate examinations in the same case. The median age was 70 (range, 18-82) years in the patient population. Metastatic castration-resistant prostate cancer (CRPC) was the most common (n = 31), followed by metastatic germ cell tumor (n = 4), small cell carcinoma of the bladder (n = 3), retroperitoneal sarcoma (n = 2), metastatic penile cancer (n = 1), adrenal small cell carcinoma (n = 1), adrenal cortical cancer (n = 1), and epithelioid renal angiomyolipoma (n = 1). A detailed breakdown is shown in Table 1.

**Table 1:** Baseline demographics and patient characteristics

	N = 44
Median age, years (range)	70 (19–82)
Sex	
Male	39 (88.6)
Female	5 (11.4)
*Prior lines of therapy for recurrent/metastatic disease at examination	
1	7 (14.3)
2	7 (14.3)
3	16 (32.7)
≥4	19 (38.8)
Cancer type of primary diagnosis	
Castration-resistant prostate cancer	31 (70.5)
Germ cell tumor	4 (9.1)
Small cell carcinoma of the bladder	3 (6.8)
Retroperitoneal sarcoma	2 (4.5)
Penile cancer	1 (2.3)
Adrenal small cell carcinoma	1 (2.3)
Adrenal cortical carcinoma	1 (2.3)
Epithelioid angiomyolipoma	1 (2.3)

As for the tissues affected, 32 were primary sites and 12 were metastatic sites. Regarding specimen collection methods, core needle biopsy was the most common method, with 28 cases, including 12 involving the removal of the primary tumor, 1 involving the removal of a metastatic tumor, and 3 involving transurethral resection. The median time from specimen collection to the notification of results was 14 months (Table 2).

**Table 2:** Details of the evaluation of dMMR mutation dMMR, mismatch repair gene deficiency

	N = 44
Tissues used for dMMR evaluation	
Primary site	32 (72.7)
Metastatic site	12 (27.3)
Specimen collection method	
Core needle biopsy	28 (63.6)
Tumor removal	13 (29.5)
Primary site	12 (27.3)
Metastatic site	1 (2.3)
Transurethral resection of the tumor	3 (6.8)
Time from specimen collection to notification of results, months (range)	14 (0–180)

**Figure 1:**
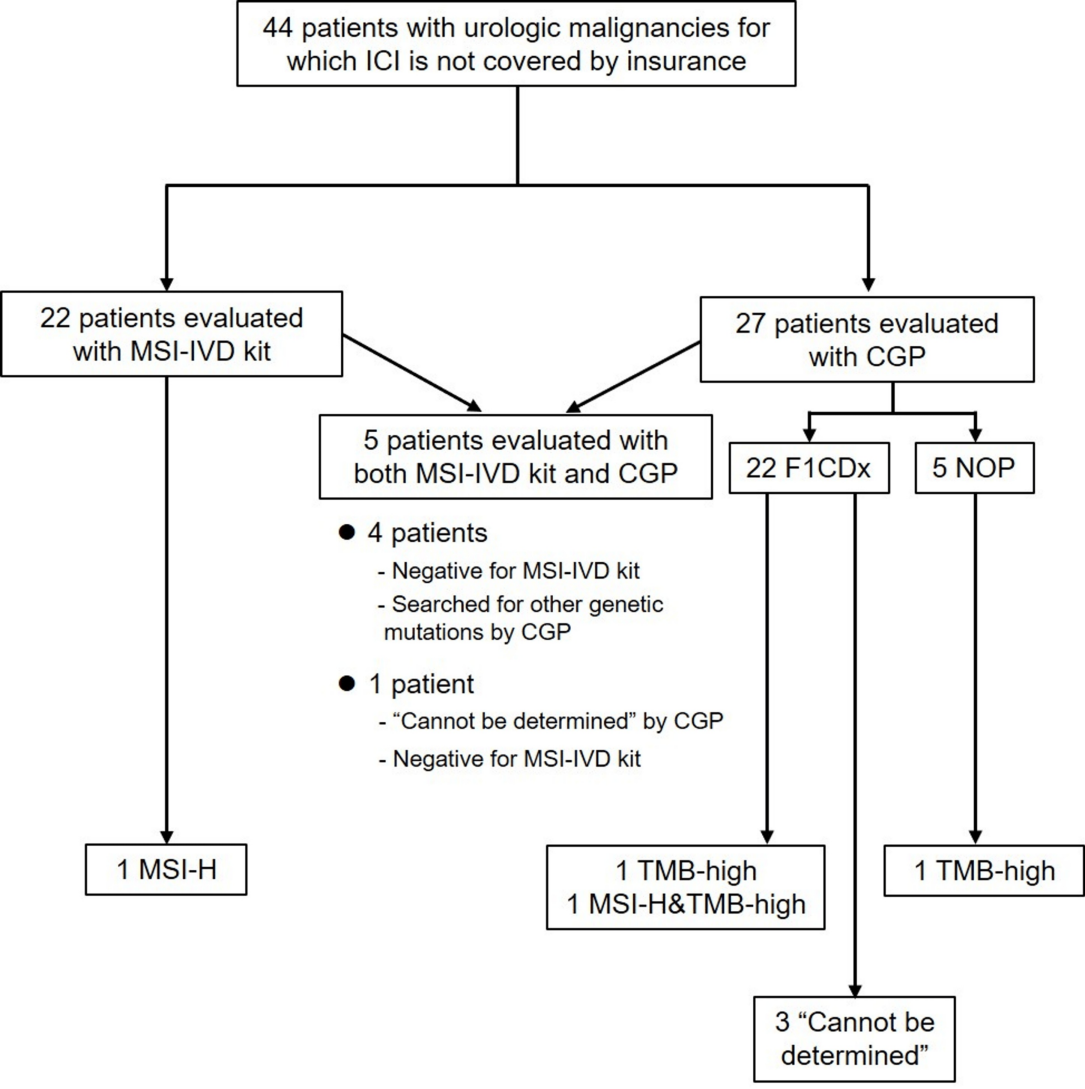
Mismatch repair gene deficiency analysis flowchart and results CGP, comprehensive genomic profiling; CRPC, castration-resistant prostate cancer; F1CDx, FoundationOne® CDx; MSI-IVD kit, microsatellite instability in vitro diagnostic kit; MSI-H, microsatellite instability-high; NOP, OncoGuide™ NCC Oncopanel System; TMB-high, high tumor mutation burden

Of the 49 tests, 1 MSI-H, 2 TMB-high, and 1 simultaneous MSI-H/TMB-high were detected, with detection rates of 4.1% and 11.1% for MSI-H and TMB-high, respectively. Ignoring duplications within the same case, two cases of MSI-H and three cases of TMB-high were detected. The MSI‐IVD kit was applied to a total of 22 cases, of which only 1 MSI-H was detected. The same patient was tested twice with the MSI-IVD kit and five with CGP. Four patients were tested twice because they were negative for the MSI-IVD kit, and CGP was used to search for other genetic mutations; one “cannot be determined” by CGP, so an additional MSI-IVD test was performed, with a negative result. In total, 27 cases were reviewed by CGP (22 F1CDx, 5 NOP), and TMB-high was detected in 2 cases and MSI-H and TMB-high simultaneously in 1 case. Of the three cases in which TMB-high was detected, two were tested by F1CDx, whereas the other was by NOP. In cases where MSI-H and TMB-high were detected simultaneously, F1CDx was performed. Of the 27 cases examined by the CGP, 3 were “cannot be determined” with respect to microsatellite status, and in all 3 cases, F1CDx was used for determination. Of the three patients who were deemed to have an undeterminable microsatellite status, one was reevaluated using an MSI‐IVD kit and was found to have a stable microsatellite status. The other two patients' general condition was deteriorating, and they were not further evaluated. In the cases evaluated in the NOP, all cases were not found to be unable to determine microsatellite stability. A detailed breakdown is shown in Figure 1.

Two patients who had MSI-H were excluded from Lynch syndrome as a result of the evaluation of their next of kin based on the Amsterdam criteria II [11]. A patient having CRPC with MSI-H detected by the MSI-IVD kit also had neuroendocrine differentiation and achieved a complete response to pembrolizumab, which persisted for a long time without any immune-related adverse events [12].

Of the three patients with TMB-high, two also had concurrent *BRCA2* mutations. Both received sequential pembrolizumab and olaparib, a poly (ADP-ribose) polymerase (PARP) inhibitor.

The duration of response to pembrolizumab was shorter in patients with a patient with TMB-high, and a CRPC patient with simultaneously detected MSI-H/TMB-high had to discontinue pembrolizumab early due to immune-related adverse events.

For another patient with CRPC in which TMB-high was detected by NOP, pembrolizumab administration is pending for now because the patient is responding to the current treatment. A summary of these four cases is presented in Table 3.

**Table 3:** Details on pembrolizumab administration in cases of dMMR detection dMMR, mismatch repair gene deficiency; CGP, comprehensive genomic profiling; MSI-H, microsatellite instability-high; TMB-high, high tumor mutation burden; CRPC-NE, castration-resistant neuroendocrine prostate cancer; CRPC, castration-resistant prostate cancer

Patient	Age	Primary disease	Prior lines of therapy	Kit used for dMMR evaluation	Types of detected dMMR	Other genetic mutations detected on CGP	Time to next line of therapy (months)	Immune-related adverse events
1	65 y	CRPC-NE	3	MSI-IVD	MSI-H	N/A	N/A	None
2	73 y	CRPC	3	F1CDx	TMB-high	BRCA2 L1908fs*2, PTEN D58fs*7	1.5	None
3	67 y	CRPC	2	F1CDx	MSI-H, TMB-high	BRCA2 T3033fs*11, ATM K2811fs*46, CHEK1 T226fs*14, ARID1A D1850fs*33, PIK3CA G122D/R93Q/D350G, PTEN T319fs*1/R173H/V290fs*1	1.5	Interstitial pneumonia (CTCAE v5.0 grade 3)
4	77 y	CRPC	3	NOP	TMB-high	Not detected	N/A	N/A

## Discussion

This study demonstrates the detection rate of MSI-H and TMB-high in urologic malignancies for which ICI therapy is not covered by Japanese insurance, an area not yet reported. The frequency of dMMR detection in prostate cancer ranges from 3% to <10% [4,6], which is consistent with the frequency of detection in this study. Similarly, the detection rate of MSI-H by the MSI-IVD kit was 4.5%, which is not high. However, the patient with CRPC having neuroendocrine differentiation who also had MSI-H detected by the MSI-IVD kit responded dramatically to pembrolizumab and achieved a long duration of response [12]. Unfortunately, the MSI-IVD kit does not allow for close examination of which gene mutations in the dMMR series were present, and this is a drawback of the MSI-IVD kit.

Furthermore, regarding MSI determination by F1CDx, a document issued by the U.S. FDA stated the following: “For patients with solid tumors whose samples have MSI scores >0.0041 and <0.0124, an MSI “cannot be determined” result is reported. Patients with this result should be retested with a validated orthogonal (alternative) method because these MSI scores represent a range of scores with low reliability [13]. Therefore, unless the patient is in poor general condition, reevaluation using the MSI-IVD kit should be considered within the scope of insurance reimbursement when the microsatellite stability status is “cannot be determined.” Ideally, evaluation by not only an MSI-IVD kit but also another CGP should be considered. However, in Japan, CGP is limited to once in a lifetime and only after completion of standard treatment for insurance purposes; thus, evaluation by another CGP is not realistic.

Recently, a study reported no significant difference between TMB status and overall survival in some cancer types [14], and although the number of cases is not that large, even in prostate cancer, CD8-positive T cells do not correlate with the neoantigen load, and the objective response rate in TMB-high tumors was lower than that in TMB-low tumors. In addition, the objective response rate for TMB-high tumors was lower than that for TMB-low tumors [15]. Conversely, multiomics analysis in prostate cancer has recently reported that high stemness as a cluster is associated with high TMB and a good response to immunotherapy if high stemness is improved by immunotherapy [16]. Therefore, whether a TMB-high of ≥10 mut/Mb, uniformly defined as a requirement for insurance approval, should be the criterion for pembrolizumab therapy in urologic malignancies requires scrutiny with attention to real-world clinical data in the future.

In this study, two cases of concurrent dMMR series mutations and *BRCA2* mutations were observed in CRPC cases. Still, no consensus has been reached on whether pembrolizumab or a PARP inhibitor should be given first in such cases because of the limited cases of cooccurrence of dMMR series mutations and *BRCA* mutations. However, PARP inhibitor-mediated unrepaired DNA damage may modulate the tumor immune microenvironment and promote responsiveness to ICI through various molecular and cellular mechanisms, such as increased genomic instability, activation of immune pathways, and programmed death-ligand 1 (PD-L1) expression on cancer cells [17], and some studies have supported the use of combination therapy with PARP inhibitor and ICI [18]. Indeed, in metastatic CRPC, the combination of olaparib and durvalumab, an anti-PD-L1 antibody, produced PSA responses (≥50% reduction) in 8 of 17 patients (47%) (NCT02484404) [19]. However, whether the efficacy and safety of combination therapy with PARP inhibitors and ICIs in MSI-H/TMB-high and *BRCA*-positive CRPC are acceptable has not yet been demonstrated and awaits further validation.

There are several limitations in the present study. First, MSI-H or TMB-high was detected only in CRPC in all cases. However, the proportion of CRPC cases in this retrospective cohort was high, so selective bias may have been introduced. Urologic malignancies for which ICIs other than CRPC are not covered by Japanese insurance belong to the so-called rare malignancies, and the true dMMR frequency needs to be verified in larger studies.

Furthermore, we would like to statistically analyze the details of the data if the number of cases was sufficient but unfortunately, the number of cases in this cohort was not sufficient, so we were unable to perform a statistical analysis.

## Conclusions

Despite the potential benefit of pembrolizumab, MSI-H or TMB-high was detected less frequently in urologic malignancies for which ICI therapy is not covered by insurance, and pembrolizumab was not successful in TMB-high cases. Thus, narrowing down the number of cases of CGP at the preliminary stage is necessary. While this study provides important findings, its findings are only preliminary. To evaluate the frequency of dMMR detection in urologic malignancies for which ICI therapy is not covered by Japanese insurance, these findings should be validated by larger and more diverse studies before generalization.
